# Duration of Protection against Malaria and Anaemia Provided by Intermittent Preventive Treatment in Infants in Navrongo, Ghana

**DOI:** 10.1371/journal.pone.0002227

**Published:** 2008-05-21

**Authors:** Matthew Cairns, Ilona Carneiro, Paul Milligan, Seth Owusu-Agyei, Timothy Awine, Roly Gosling, Brian Greenwood, Daniel Chandramohan

**Affiliations:** 1 Department of Epidemiology and Population Health, London School of Hygiene & Tropical Medicine, London, United Kingdom; 2 Department of Infectious and Tropical Diseases, London School of Hygiene & Tropical Medicine, London, United Kingdom; 3 Kintampo Health Research Centre, Kintampo, Ghana; 4 Navrongo Health Research Centre, Navrongo, Ghana; Mahidol University, Thailand

## Abstract

**Background:**

Intermittent preventive treatment for malaria in Infants (IPTi) has been shown to give effective and safe protection against malaria. It has been suggested that IPTi might have long-lasting beneficial effects but, in most settings, the protection provided by IPTi appears to be short-lived. Knowledge of the duration of protection given by IPTi would help interpret the results of existing trials and suggest optimal delivery schedules for IPTi. This study investigated how the protective efficacy of IPTi against malaria and anaemia changes over time.

**Methods and Findings:**

A secondary analysis of data from a cluster-randomised, placebo-controlled trial of IPTi using sulfadoxine-pyrimethamine (SP) in Ghana was conducted. In this trial IPTi was given to 2485 infants at 3, 4, 9 and 12 months of age; children remained in follow-up until two years of age. Poisson regression with a random effect to adjust for the cluster-randomised design was used to determine protective efficacy of IPTi against clinical malaria and anaemia in defined time strata following administration of IPTi. Analysis of first-or-only clinical malaria episode following the individual IPTi doses showed that some protection against malaria lasted between 4 to 6 weeks. A similar pattern was seen when the incidence of all malaria episodes up to 2 years of age was analysed in relation to the most recent IPT, by pooling the incidence of malaria after the individual IPTi doses. Protective efficacy within four weeks of IPTi was 75.2% (95% CI: 66–82) against malaria, 78.9% (95% CI: 69–86) against high parasite density malaria, and 93.8% (95% CI: 73–99) against anaemia. Protection against these outcomes was short-lived, with evidence of any effect lasting for only 6, 6 and 4 weeks respectively. Protection in children who were parasitaemic when receiving IPTi appeared to be of shorter duration than in uninfected children. There was no evidence of any benefit of IPTi after the immediate period following the IPTi doses.

**Conclusions:**

Intermittent preventive treatment provides considerable protection against malaria and anaemia for short periods, even in an area of intense seasonal transmission. Due to the relatively short duration of protection provided by each dose of IPTi, this treatment will be of most benefit when delivered at the time of peak malaria incidence.

## Introduction

Intermittent preventive treatment (IPT) is a promising strategy for preventing malaria morbidity in infants and children, particularly in sub-Saharan Africa where the disease burden is highest. Sulfadoxine-pyrimethamine (SP) given to Tanzanian infants alongside routine vaccinations gave high protective efficacy against malaria and anaemia; this protection appeared to be sustained long after the chemoprophylactic period of SP, suggesting that development of protective immunity might have been facilitated by the treatment [1]–[3]. Subsequent studies in infants and children have shown IPT to be an effective preventive measure against malaria, but none of these have shown a sustained benefit beyond the period that might reasonably be attributed to drugs [4]–[12]. The most likely explanation is that IPT reduces the incidence of malaria primarily through clearing existing parasitaemia (or reducing it to a level below the fever threshold) and preventing new infections sensitive to the drug used for IPT [13].

Although there is now a large body of evidence that three courses of IPT given alongside Expanded Programme on Immunisation (EPI) vaccines can reduce the incidence of malaria in infants, the optimum timing and frequency of IPT doses is still debated [14]. The EPI-linked IPT schedule was based on the feasibility of delivering IPT rather than on maximising its potential benefits. Data on the duration of protection following each dose of IPT would help to define the optimum interval between IPT doses, enabling development of the optimal delivery strategy for a given location. Knowledge of how long IPT gives protection for might also explain the differences in efficacy observed when different delivery strategies for IPT have been used. For example, four doses of IPT delivered to infants through the EPI in Ghana achieved a protective efficacy (PE) of 24.8% (95% confidence interval (CI) 14.3–34.0) over a twelve-month period [4], whereas monthly IPT given to children under 5 in Senegal over a three-month period achieved protective efficacy of 86%; (95% CI: 80–90) [5].

## Methods

This study used data from a cluster randomized trial of IPTi in Navrongo, Ghana described in detail elsewhere [4]. In brief, four doses of SP or placebo were given to infants at 3, 4 and 9 months of age at time of vaccinations delivered through the EPI, and at the 12 month growth-monitoring visit. Individual doses of IPTi are referred to as IPT-1, IPT-2, etc. Primary endpoints in the trial were clinical malaria (history of fever or temperature ≥37.5°C plus malaria parasites detected on a blood smear) detected in children attending health centres or the hospital and anaemia (packed cell volume <24%) in children admitted to the study hospital for any illness; the same endpoints have been used for this analysis. High parasite density malaria episodes as defined in the trial (malaria with parasite density ≥5000 parasites/µl) were also examined.

Covariates pre-specified for adjustment in analysis of the main trial were sex, urban residence and mosquito net usage. Reported bednet use among study children was low (13%) and insecticide treated net use very low (2%); there were no data for 26% of children. For this reason bednet use was not included in the models. The primary analysis was performed unadjusted for covariates; the effect of adjusting for age at time of dose, sex and place of residence (rural or urban) was then explored.

### Protective efficacy of individual IPT doses

Person time at risk of malaria was calculated from the date of a given dose of IPTi until a subsequent dose was administered, malaria was diagnosed, or the individual exited the study (dropped out, migrated or died), whichever occurred first. Kaplan-Meier failure plots were used to examine incidence of malaria after each IPTi dose until the following IPTi dose or until the end of follow up. A poisson regression model was fitted with a gamma distributed random effect to account for potential correlation within clusters of children. Lexis expansion [15] was used to examine malaria incidence in defined strata following each dose of IPTi, allowing week-specific estimates of protective efficacy (1-Incidence rate ratio, IRR (SP versus placebo)%) to be calculated. Evidence for interaction between time since receiving IPTi and protective efficacy was assessed using the likelihood ratio test (LRT), initially without adjusting for covariates, and after including the covariates age, sex and place of residence (rural or urban) in the model.

### Protective efficacy against all malaria and anaemia episodes up to two years of age

Person time at risk of malaria and anaemia was calculated from first dose of IPT until the child exited the study or reached two years of age. Lexis expansion was performed and a variable was created that showed the number of weeks since the most recent treatment throughout follow-up. This allowed incidence of any malaria or anaemia episode that occurred between the first treatment and two years of age to be related to time since most recent IPTi dose; pooling the analysis across doses enabled a more robust analysis of protective efficacy over time. For malaria and high parasite density malaria, follow-up was stratified by week since IPT; there were fewer episodes of anaemia so two-week strata were used. Incidence rate in a given time stratum was compared between trial arms using a poisson regression model with a cluster-level random effect.

Participants who had received chloroquine or quinine, the drugs of choice during the study period, for the treatment of clinical episodes of malaria were not considered to be free of the risk of malaria during the post treatment period. This approach gives a more pragmatic estimate of the effect of IPTi, by making a direct comparison between IPTi and routine case-management of malaria. Consequently, our estimate of the protective efficacy of IPTi may be slightly more conservative than that reported by other studies that deducted 3 to 4 weeks post treatment for each episode of malaria to account for the prophylactic effect of the antimalarial treatment.

To investigate if protection in Navrongo was compatible with that reported from studies that have used monthly IPT, an analysis that only included episodes that occurred within four weeks of an IPTi dose was performed.

### Effect of parasitaemia on protective efficacy of IPTi

In addition to providing prophylaxis, IPT should in most cases clear parasitaemia present at the time of treatment. This could be beneficial since many children in this age group will not have developed sufficient immunity to prevent an existing infection increasing in density and causing disease [16]. However, in children carrying low density infections that do not cause illness, clearance of parasitaemia may not be advantageous as it could result in the loss of cross-protective immunity against similar parasite genotypes (premunition) and consequently increase the risk of subsequent malaria [17], [18]. To investigate this, protective efficacy of IPT in children with and without microscopically detected parasitaemia at time of IPT dose was compared.

The approach taken was the same as for the pooled analysis of all malaria episodes: a poisson regression model was fitted to the data and follow-up time stratified by week since most recent treatment. Blood smears were not taken at the dose given at four months of age (scheduled as IPT-2) but were available for 106 individuals who received IPT-2 at a later date due to vomiting or absence at the scheduled time. Follow-up time after doses at which parasitaemia was not determined was excluded from this analysis.

All analyses were performed in Stata version 9 (StataCorp, College Station, Texas).

### Ethical Approval

Ethical approval for this study was granted by the Ethics Review Board of the London School of Hygiene and Tropical Medicine, number 05/176.

## Results

### Protective efficacy of individual IPT doses

**Figure 1:**
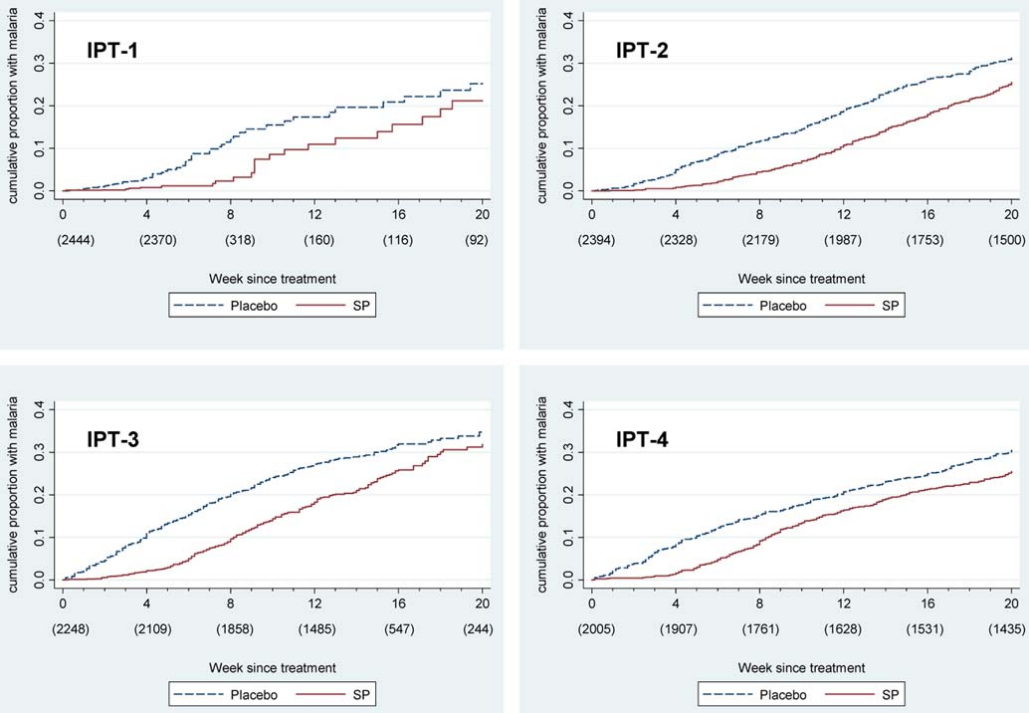
Kaplan-Meier failure plots for individual IPT doses. Kaplan-Meier plots showing cumulative proportion of children with a malaria episode following IPT doses 1–4. Numbers below x-axis labels indicate number of children remaining in follow-up at that time point.

Kaplan-Meier failure plots for clinical malaria following individual IPTi doses are shown in figure 1. Length of follow-up time was unrestricted in this analysis since the interval between doses could be longer than scheduled if participants did not receive a subsequent dose or received it late, but only the first 20 weeks following treatment are presented. In the survival analysis of the protective effect of IPT-1, most children received IPT-2 after 28 days. Consequently, single episodes of malaria later in the survival curve have a large effect because the number remaining at risk is small.

**Figure 2:**
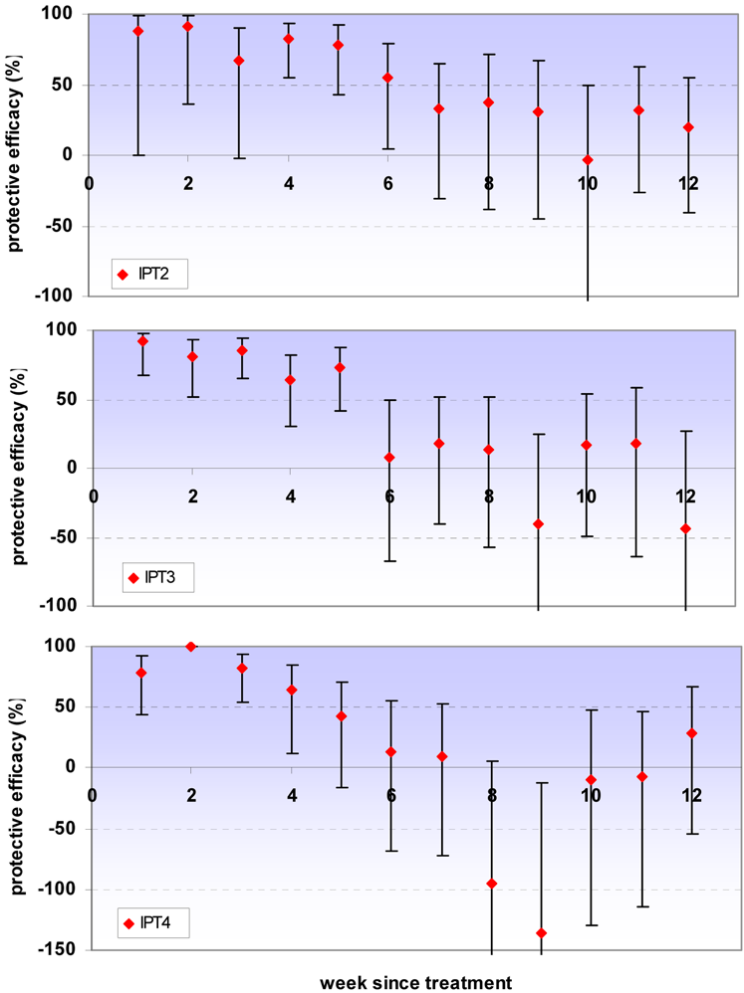
Protective efficacy of individual IPT doses against clinical malaria. Protective efficacy against first-or-only episode of clinical malaria (history of fever or temperature ≥37.5°C plus malaria parasites detected on a blood smear) by week since treatment for IPTi doses 2, 3 & 4. Error bars indicate 95% confidence intervals. The y-axis is truncated at −100 for IPT2 and IPT3, and at −150 for IPT4. No children given SP had malaria during week 2 after IPT4.

Poisson regression with a random effect to adjust for the clustered design was used to estimate protective efficacy against clinical malaria with increasing time since IPTi doses. Adjusting for age, sex and place of residence of children made negligible changes to the estimates of protective efficacy; data are presented from the unadjusted models. There was strong evidence of an interaction between IPT efficacy and time since IPT dose (LRT p<0.01 in all analyses) suggesting that protective efficacy depends on how long ago the IPT dose was received. Protection is high initially but then appears to be lost rapidly. Consistent with other IPTi studies, protective efficacy is presented up to twelve weeks (3 months) post dose. No evidence of either protective or detrimental effects was observed after this period for any of the doses of IPTi. For all IPT doses there is evidence of significant protection for at least four weeks, protection appears to last for at least five weeks after IPT-3 and there is weak evidence of some protection up to 6 weeks for IPT-2 (table 1 & figure 2). This analysis was not possible for IPT-1 since this was followed in most cases by IPT-2 28 days later and incidence of malaria was low even among children who received placebo in this age group.

**Table 1:** Protective efficacy of individual IPT doses against clinical malaria.

	Protective Efficacy against clinical malaria (95% Confidence Interval)		
IPT-1			IPT-2
IPT dose	IPT-2	IPT-3	IPT-4
**Week since IPT**			
1	88 (0, 98)	92 (68, 98)	79 (43, 92)
2	92 (36, 99)	81 (51, 93)	100*
3	67 (−2, 90)	85 (65, 94)	83 (54, 93)
4	83 (55, 93)	64 (30, 82)	64 (12, 85)
5	78 (42, 92)	74 (41, 88)	42 (−16, 71)
6	55 (4, 79)	8 (−67, 49)	14 (−68, 56)
7	33 (−31, 65)	18 (−40, 52)	9 (−72, 52)
8	37 (−38, 71)	13 (−57, 52)	−95 (−302, 5)
9	31 (−45, 67)	−41 (−165, 25)	−136 (−394, −13)
10	−3 (−109, 49)	17 (−49, 53)	−9 (−130, 48)
11	31 (−27, 63)	18 (−64, 59)	−7 (−114, 47)
12	20 (−41, 55)	−44 (−181,27)	29 (−54, 67)

Protective efficacy against first-or-only episode of clinical malaria (history of fever or temperature ≥37.5°C plus malaria parasites detected on a blood smear) by week since treatment for IPTi doses 2, 3 & 4.

* No children given SP had malaria during week 2 after IPT4.

### Protective efficacy against all malaria and anaemia episodes up to two years of age

**Figure 3:**
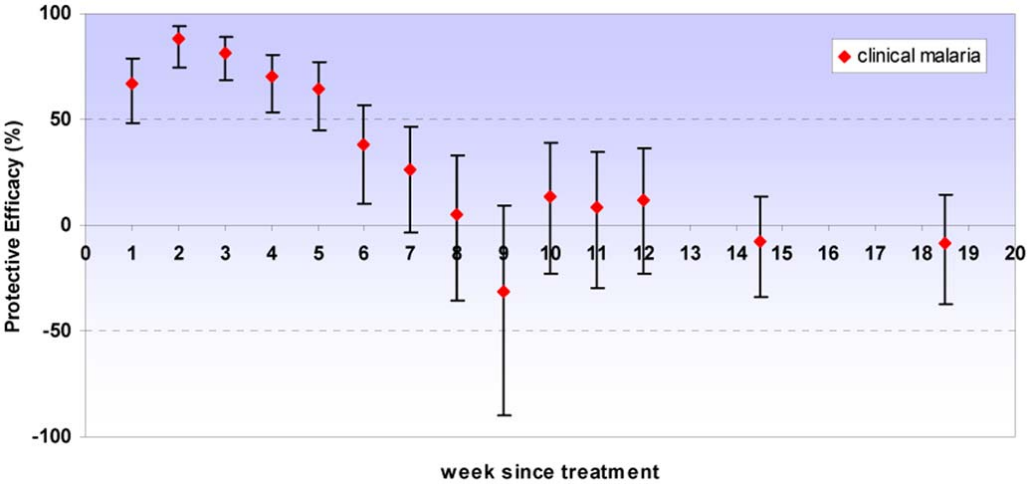
Protective efficacy of IPT against clinical malaria. Protective efficacy of IPTi by week since treatment against clinical malaria (history of fever or temperature ≥37.5°C plus malaria parasites detected on a blood smear). Error bars indicate 95% confidence intervals. PE estimates are for all IPT doses combined; all episodes that occurred before two years of age were included in the analysis. No long term protection was observed; for brevity estimates are presented up to twenty weeks following treatment. Incidence in weeks 13–16 and 17–20 was aggregated; data points are shown at the midpoint of each interval.

There was strong evidence of a high protective effect against all episodes of clinical malaria and high parasite density malaria in the first four to five weeks following IPT. Some protection was seen up to six weeks but from seven weeks onwards there was no evidence of any remaining benefit of having received IPTi. Protection against anaemia was high up to four weeks after IPT. No evidence of either a protective or detrimental effect was observed after the immediate period following IPTi for any of these outcomes. Our analysis included all of the follow-up period to two years of age, but since no protective effect was observed except for the immediate period following IPT, graphs are truncated at 20 weeks post-dose (figures 3–5, table 2). Excluding the first week after IPT, the point estimate of protective efficacy declines with each successive week until efficacy approaches zero. Adjusting for age, sex and place of residence of children again had a negligible effect on the estimates of protective efficacy; data are presented from the unadjusted models.

**Figure 4 pone-0002227-g004:**
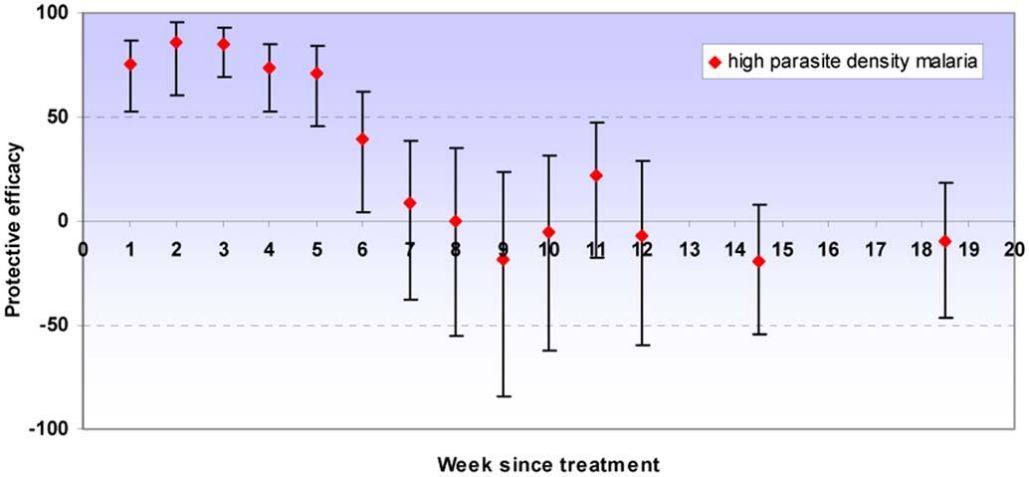
Protective efficacy of IPT against high parasite density malaria. Protective efficacy of IPTi by week since treatment against high parasite density malaria (clinical malaria with parasite density ≥5000/µl). Error bars indicate 95% confidence intervals. PE estimates are for all IPT doses combined; all episodes that occurred before two years of age were included in the analysis. No long term protection was observed; for brevity estimates are presented up to twenty weeks following treatment. Incidence in weeks 13–16 and 17–20 was aggregated; data points are shown at the midpoint of each interval.

**Figure 5:**
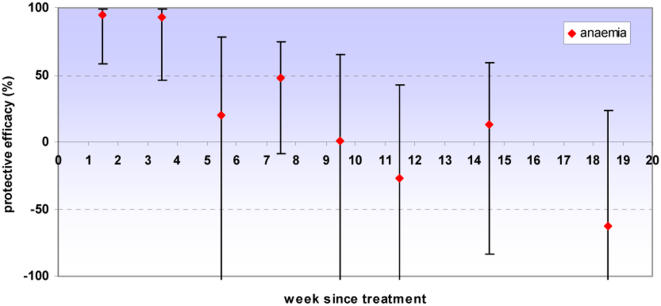
Protective efficacy of IPT against anaemia. Protective efficacy of IPTi by week since treatment against anaemia (packed cell volume <24%). Error bars indicate 95% confidence intervals. PE estimates are for all IPT doses combined; all episodes that occurred before two years of age were included in the analysis. No long term protection was observed; for brevity estimates are presented up to twenty weeks following treatment. Weeks were aggregated as 1–2, 3–4, 5–6, 7–8, 9–10, 11–12, 13–16 & 17–20 weeks since treatment; data points are shown at the midpoint of each interval. The y-axis is truncated at −100, for full data see table 2.

**Table 2:** Protective efficacy of IPT against clinical malaria, high parasite density malaria and anaemia.

	Protective Efficacy (95% confidence interval)		
Endpoint	Clinical malaria	High parasite density malaria	Anaemia
**Week since most recent IPTi**			
1	66.6 (48, 78)	75.1 (53, 87)	
2	88.0 (75, 94)	86.3 (61, 95)	94.5 (58, 99)
3	81.3 (69, 89)	84.9 (69, 93)	
4	70.2 (54, 81)	73.5 (53, 85)	92.9 (46, 99)
5	64.5 (45, 77)	70.9 (45, 85)	
6	37.9 (10, 57)	39.7 (4, 62)	20.3 (−198, 79)
7	25.9 (−4, 47)	8.5 (−37, 39)	
8	5.1 (−35, 34)	−0.4 (−55, 35)	48.0 (−9, 75)
9	−31.4 (−90, 9)	−18.3 (−84, 24)	
10	13.6 (−23, 39)	−5.4 (−62, 32)	1.0 (−183, 65)
11	8.2 (−30, 35)	21.5 (−18, 48)	
12	11.7 (−23, 37)	−6.7 (−60, 29)	−26.6 (181, 43)
16	−7.6 (−34, 14)	−19.1 (−54, 8)	13.2 (−84, 59)
20	−8.1 (−37, 15)	−9.7 (−47, 18)	−62.7 (−247, 24)

Protective efficacy of IPTi by week since treatment for all IPT doses combined. Protective efficacy is shown against clinical malaria (history of fever or temperature ≥37.5°C plus malaria parasites detected on a blood smear), high parasite density malaria (clinical malaria with parasite density ≥5000 parasites/µl) and anaemia (packed cell volume <24%). All episodes that occurred before two years of age were included in the analyses. No long term protection was observed; for brevity estimates are presented up to twenty weeks following treatment. Fewer episodes of anaemia made it necessary to stratify follow-up every two weeks for this endpoint.

To compare the effect of our more pragmatic approach with those of previous papers, we repeated the analysis removing 28 days of person-time at risk after a clinical malaria episode. The results were remarkably similar to those presented, although there was evidence of a small amount of protection against clinical malaria lasting for an additional week after IPTi (i.e. into the seventh week after treatment rather than the sixth week). After this period there remained no evidence of any protection.

### Comparison with seasonal IPT trials

Considering only incidence within the first four weeks following a dose of IPT, protective efficacy was high: 75.2% (95% CI: 66–82) against malaria, 78.9% (95% CI: 69–86) against high parasite density malaria, and 93.8% (95% CI: 73–99) against anaemia.

### Effect of parasitaemia on protective efficacy of IPTi

**Figure 6:**
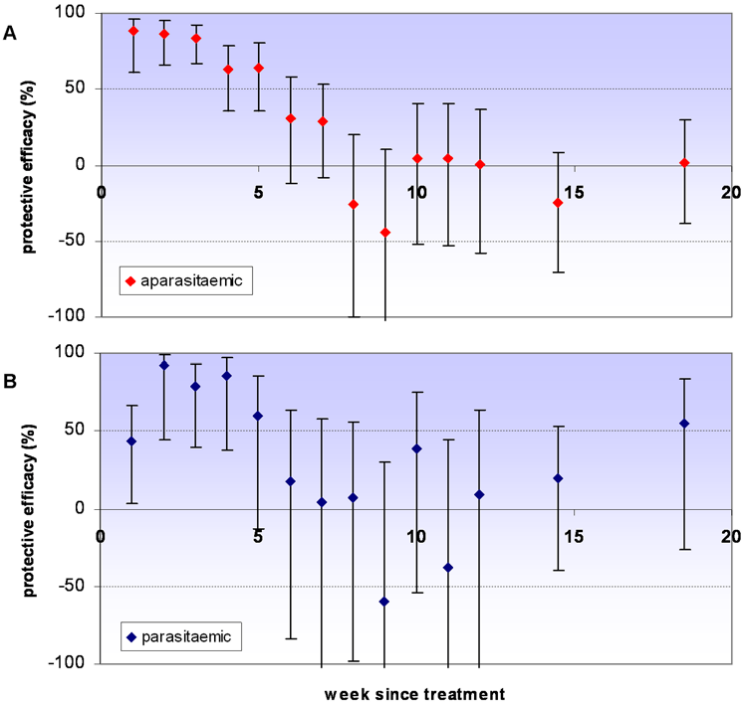
Effect of parasitaemia on protective efficacy following IPT. Protective efficacy against clinical malaria (history of fever or temperature ≥37.5°C plus malaria parasites detected on a blood smear) with time in children aparasitaemic (A) and parasitaemic (B) at time of IPT. Error bars indicate 95% CIs. PE estimates are for all IPT doses combined; all episodes that occurred before two years of age after an IPT dose at which a blood slide was taken were included. No long term protection was observed; for brevity estimates are presented up to twenty weeks following treatment. Incidence in weeks 13–16 and 17–20 was aggregated; data points are shown at the midpoint of each interval. For clarity of presentation the y-axis is truncated at −100%.

Individuals who are parasitaemic when given IPT experience a higher incidence of malaria in the short term, but this risk wanes with time. Within four weeks of treatment the incidence rate for clinical malaria was 2.66 times greater in children who were initially parasitaemic (95% CI: 2.12 to 3.34). There was no evidence of interaction between parasitaemia and the overall protective efficacy of IPT (LRT p = 0.6521). However, there was strong evidence of interaction between parasitaemia at time of dose and the protective efficacy of IPT over time (LRT p<0.001). Adjusting for age, sex and place of residence of children made negligible changes to the estimates of protective efficacy; data are presented from the unadjusted model. In children parasitaemic at the time of IPT, protective efficacy is lower in the first week, but is then high in the period up to 5 weeks following the IPTi dose. There is a suggestion that protection may be lost faster in children initially parasitaemic than in children without parasites (figure 6).

## Discussion

### Duration of protection given by IPTi

The protection provided by IPTi in Navrongo was of limited duration. Protective efficacy against malaria and high parasite density malaria was high during the first four to five weeks after treatment, retaining some efficacy up to six weeks; there was no evidence of a protective effect from week seven onwards. Protection against anaemia lasted for four weeks following treatment. Since periods when children were being treated for malaria were not removed from time at risk in this analysis, our estimate of protective efficacy may be slightly conservative and the duration of protection slightly underestimated because more episodes of malaria occurred in the placebo group. However, removing 28 days person-time at risk after each malaria episode increased the duration of protection following IPTi by just one more week and there was no evidence of any protection after week seven post IPTi. The period of highest protection was still within the first four to five weeks. We do not therefore consider that our conservative approach has resulted in any important underestimation of the duration of protection given by IPTi.

There is weak evidence of an elevated incidence of malaria in the SP group relative to those on placebo in weeks eight and nine following IPT-4. Increased malaria incidence eight to ten weeks after treatment with SP and artesunate was also observed in a mass-drug administration trial in the Gambia [19]. However, this effect was not seen in an analysis when multiple episodes of malaria were considered rather than first-or-only episode (data not shown) suggesting that this may be an artefact of frailty effects in our survival analysis rather than rebound [20]. In other words, children in the placebo group at high risk of malaria are likely to have had malaria and exited the first-or-only episode analysis by the time protection has worn off in the children given SP. This means that the comparison at later time points is between almost all children given SP (some of who are at high risk) and only the placebo children at low risk of malaria. Even if this was a true instance of minor rebound morbidity following IPT, it does not appear to outweigh the episodes prevented by earlier protection.

The plasma half-lives of sulfadoxine and pyrimethamine are between 5–11 days and 3–5 days respectively in adults, but there are no data on pharmacokinetics in infants [13], [21]–[23]. Direct comparison of pharmacokinetic data with respect to the protection observed is further hindered by the lack of an established minimum inhibitory concentration *in vivo*. What can be expected is that the direct parasiticidal effect of treatment cannot last indefinitely, and one would expect a gradual reduction in protective efficacy which accelerates over time, as seen here [23]. Watkins *et al.* have estimated that SP may be able to provide prophylaxis against fully sensitive *Plasmodium falciparum* for approximately 60 days [22]. Given that there was documented SP resistance in the area at the time of the study and the entomological inoculation rate (EIR) was high [24], [25] one would expect protection to be shorter than this, and indeed this is what was observed. A pharmacokinetic study published since the completion of this trial has shown that under-dosing of SP in children may be widely under-recognised at present [26], which could explain the apparent reduced duration of protection given by later IPTi doses in older infants.

### Comparison with seasonal IPT trials

Protective efficacy against clinical malaria within four weeks of treatment was high: 75.2% (95% CI: 66–82). IPTi in this setting therefore attains an efficacy similar to that achieved in Niakhar, Senegal with monthly IPT (86%; 95% CI: 80–90)[5] when a similar period after treatment is compared. The lower protective efficacy during the entire study period (24.8%; 95% CI 14.3–34.0) [4] is therefore a consequence of the prolonged periods between IPT doses, rather than differing efficacy of each dose of IPTi. The remaining difference in protective efficacy between these two sites could be a consequence of the Niakhar trial including children up to 5 years of age, artesunate being used in addition to SP, and the lower transmission intensity in the Senegal site (estimated EIR in Navrongo: 418 infective bites per year (2001–02) [25]; EIR in Niakhar: 10 infective bites per year [5]).

### Effect of parasitaemia on protective efficacy of IPTi

There was no evidence of an elevated incidence of malaria in children who were parasitaemic when given IPT that would suggest that clearance of parasites results in loss of cross protective immunity against similar parasite genotypes and has a cost attached. In fact it appears that infections that are asymptomatic at time of detection are likely to lead to disease in the short term in this age group and should be treated, a finding that agrees with existing literature on this subject. A study in Uganda found that asymptomatic parasitaemia was a clear risk factor for clinical malaria within thirty days of detection [27], while a recent analysis of data from eight countries in sub-Saharan Africa found that asymptomatic parasitaemia after treatment carried a substantial risk of malaria within days of discovery, particularly in young children [28].

Parasitaemic children experienced a markedly increased incidence in the first week after IPT, even those who were given SP. This effect was not age-related, so cannot be solely due to the possible under-dosing in older children. Presumably this reflects cases where the drug was given too late to prevent an incipient episode since antimalarial drugs do not reduce the parasite burden instantaneously, even if given intravenously [29]. Early treatment failures for therapeutic use of SP in Navrongo at the time of the study was 8.6% (95% CI 4.2, 15.3) [24].

For children free of parasites when they received IPT, the protective efficacy profile reflects the ‘true’ prophylactic effect of SP over time. Children who are parasitaemic are at the additional risk of recrudescence of the original infection if the parasite is resistant to SP, so protection may appear to last for less time than in uninfected children. Existing knowledge suggests that use of long acting antimalarials such as SP may result in very late recrudescence if not all parasites are killed but multiplication is suppressed until most of the drug is eliminated [23], [30]. There is a suggestion of this pattern in our results: protective efficacy for parasitaemic children falls towards zero more rapidly than in children free of parasitaemia (figure 6). We do not have data on parasite genotypes with which to differentiate true recrudescence from re-infections. An alternative explanation is that children parasitaemic at time of treatment are more exposed to malaria infection in general, so are more likely to be infected and develop malaria in the follow-up period after IPT. In the short-term period after IPT, however, the existing infection might be considered as the more likely cause of malaria, given the resistance patterns in Navrongo at the time and differing efficacy of SP as a therapeutic and prophylactic agent (discussed below) [24].

The protective efficacy curves including children with and without parasitaemia together (figures 3, 4 & 5) remain of primary interest since they show the overall protection given that some children were infected when given IPT and some were not. This will be the case in almost all situations in which IPT is used. However, identification of the possible relationship between parasitaemia and apparent duration of IPT protection may help to interpret the results of subsequent trials. The estimate of protective efficacy obtained from a trial will be a function of the extent of protection and the duration for which this protection appears to last. Consequently, protective efficacy estimates could be affected by the proportion of children infected at time of treatment as well as by levels of resistance to the antimalarial used. Increase in either of these factors would predict the duration of protection to be shorter and the estimate of protective efficacy to be smaller. Post-treatment prophylaxis may indeed be a more important component of IPT in infants than preventing recrudescence [13], [23]. However, for a given level of drug resistance, if prevalence of infection at time of treatment rises, clearance of the existing infection will increase in importance in relation to prophylaxis.

It is plausible that SP may remain effective for prophylaxis despite reduced therapeutic efficacy due to the difference in parasite burdens between a microscopically patent blood-stage infection (>10^8^ parasites) and a recently inoculated parasite on emergence from the exo-erythrocytic schizont (approximately 10^5^ parasites) [31]. If this is the case, recrudescence due to drug resistance may commonly detract from the prophylactic protection given by SP when used for IPT and partnering with another effective antimalarial could help eliminate existing high density infections and maximise the protection given. Amodiaquine is one candidate for this role, having been successfully partnered with SP for IPT in children in Senegal [12].

### Conclusion

In all analyses there was strong evidence of a beneficial effect of IPT up to four weeks after treatment and in some cases this appeared to last up to five or six weeks. The largest drop in protective efficacy against both clinical malaria and high parasite density malaria was between five and six weeks after IPT (table 2); before this time there is a relatively gradual decline in protection. This suggests that for children at periodic high risk of malaria in a seasonal transmission setting, IPT given on a monthly (or approximately monthly) basis would be better than the current option of a prolonged interval between IPT doses. The consequences of a single missed dose would also be less if IPT is repeated soon afterward. As discussed, monthly IPT has already been demonstrated as highly efficacious in Senegal and Mali [5], [9], [12], but further work is needed to address the cost and logistics of large-scale seasonal IPT.

If regular delivery of IPT during the transmission season is to become a widespread approach to controlling malaria in areas where the majority of the burden is concentrated in a few months, several issues will need to be addressed. Some of these are not new: regular intermittent treatment is akin to seasonal chemoprophylaxis [32]. Studies of seasonal IPT with longer follow-up are needed to rule out existing concerns related to this strategy, including acceleration of development of drug resistance and impairment of development of immunity before deployment on a wider scale.
